# Information needs and development of a question prompt sheet for upper extremity vascularized composite allotransplantation: A mixed methods study

**DOI:** 10.3389/fpsyg.2022.960373

**Published:** 2022-09-05

**Authors:** Jessica Gacki-Smith, Brianna R. Kuramitsu, Max Downey, Karen B. Vanterpool, Michelle J. Nordstrom, Michelle Luken, Tiffany Riggleman, Withney Altema, Shannon Fichter, Carisa M. Cooney, Greg A. Dumanian, Sally E. Jensen, Gerald Brandacher, Scott Tintle, Macey Levan, Elisa J. Gordon

**Affiliations:** ^1^Center for Health Services and Outcomes Research, Northwestern University Feinberg School of Medicine, Chicago, IL, United States; ^2^Center for Surgical and Transplant Applied Research, Department of Surgery, NYU Langone Health Transplant Institute, New York University Grossman School of Medicine, New York, NY, United States; ^3^Center for Rehabilitation Sciences Research, Uniformed Services University of the Health Sciences, Walter Reed National Military Medical Center, Bethesda, MD, United States; ^4^Henry M. Jackson Foundation, Center for Rehabilitation Sciences Research, Uniformed Services University of the Health Sciences, Walter Reed National Military Medical Center, Bethesda, MD, United States; ^5^Department of Plastic and Reconstructive Surgery, Johns Hopkins University School of Medicine, Baltimore, MD, United States; ^6^Department of Surgery, Northwestern University Feinberg School of Medicine, Chicago, IL, United States; ^7^Department of Medical Social Sciences and Department of Surgery, Northwestern University Feinberg School of Medicine, Chicago, IL, United States; ^8^Johns Hopkins University School of Medicine, Baltimore, MD, United States; ^9^Uniformed Services University of the Health Sciences, Walter Reed National Military Medical Center, Bethesda, MD, United States; ^10^Center for Surgical and Transplant Applied Research, Department of Surgery and Department of Population Health, NYU Langone Health Transplant Institute, New York University Grossman School of Medicine, New York, NY, United States; ^11^Johns Hopkins University School of Medicine, Baltimore, MD, United States; ^12^Department of Surgery-Division of Transplantation, Center for Health Services and Outcomes Research, Center for Bioethics and Medical Humanities, Northwestern University Feinberg School of Medicine, Chicago, IL, United States

**Keywords:** informed consent, patient–clinician communication, ethics, treatment decision making, patient-centered care, upper limb amputation, VCA

## Abstract

**Background:**

People with upper extremity (UE) amputations report receiving insufficient information about treatment options. Furthermore, patients commonly report not knowing what questions to ask providers. A question prompt sheet (QPS), or list of questions, can support patient-centered care by empowering patients to ask questions important to them, promoting patient-provider communication, and increasing patient knowledge. This study assessed information needs among people with UE amputations about UE vascularized composite allotransplantation (VCA) and developed a UE VCA-QPS.

**Methods:**

This multi-site, cross-sectional, mixed-methods study involved in-depth and semi-structured interviews with people with UE amputations to assess information needs and develop a UE VCA-QPS. Qualitative data were analyzed by thematic analysis; quantitative data were analyzed by descriptive statistics. The initial UE VCA-QPS included 130 items across 18 topics.

**Results:**

Eighty-nine people with UE amputations participated. Most were male (73%), had a mean age of 46 years, and had a unilateral (84%) and below-elbow amputation (56%). Participants desired information about UE VCA eligibility, evaluation process, surgery, risks, rehabilitation, and functional outcomes. After refinement, the final UE VCA-QPS included 35 items, across 9 topics. All items were written at a ≤ 6th grade reading level. Most semi-structured interview participants (86%) reported being ‘completely’ or ‘very’ likely to use a UE VCA-QPS.

**Conclusion:**

People with UE amputations have extensive information needs about UE VCA. The UE VCA-QPS aims to address patients’ information needs and foster patient-centered care. Future research should assess whether the UE VCA-QPS facilitates patient-provider discussion and informed decision-making for UE VCA.

## Introduction

Upper extremity (UE) vascularized composite allotransplantation (VCA) is a treatment option for people with hand and/or arm amputations that involves “transplantation of non-autologous vascularized tissues including skin, muscle, nerve, tendon and/or bone as a functional unit (e.g., a hand) to replace non-reconstructible tissue defects” (American Society of Transplant Surgeons, 2022). To date, 56 UE VCAs have been performed on 37 patients in the United States (OPTN, 2022), and more than 120 have been performed worldwide (Shores et al., 2017).

Upper extremity VCA is one of several treatment options (e.g., myoelectric and mechanical prostheses) for people with UE amputations. UE VCA is an innovative treatment modality that restores body wholeness and function for patients with complex reconstructive needs (Rose et al., 2019). Evidence suggests that people with UE amputations lack awareness and knowledge of UE VCA. People with UE limb loss report receiving insufficient information about their treatment options and report that healthcare providers do not engage in enough discussion about their condition (Nielsen, 1991; Pedlow et al., 2014; Pasquina et al., 2015; Bennett, 2016). Further, little is known about what information people with UE amputations need to know about UE VCA, which is necessary for optimizing their informed consent.

A patient-centered approach to care involves respecting and responding to patients’ needs and preferences, so they can make informed treatment decisions (Institute of Medicine, Committee on Quality of Health Care in America, 2001; Epstein and Street, 2011). Elements of patient-centered care include effective communication and information exchange. Effective communication entails a dialogue between provider and patient, and patient question-asking can increase patient engagement, empowerment, and the quality of provider information-giving (Barnlund, 1970; Shepherd et al., 2011; Barton et al., 2020). However, patients commonly do not know what questions to ask to guide decision making (Lopez-Vargas et al., 2014; Lederer et al., 2016; Schwarze et al., 2020). To date, no educational interventions have been developed to increase knowledge and understanding about UE VCA for people with UE amputations.

Communication tools, such as a question prompt sheet (QPS), can facilitate patient-provider communication and support patient-centered care (Belkora et al., 2009; Gordon and Ison, 2014; Brandes et al., 2015; Sansoni et al., 2015; Satteson et al., 2020). A QPS is a list of questions that can empower patients to ask questions about topics important to them and promote discussion between patients and their providers. QPSs have been shown to help patients obtain information, increase the number of questions asked, improve patient-provider communication, increase patient knowledge, increase patient satisfaction, and reduce or have no effect on consultation time across clinical settings (e.g., oncology, chronic kidney disease, palliative care) (Brown et al., 2001, 2011; Gaston and Mitchell, 2005; Brandes et al., 2015; Sansoni et al., 2015; Arthur et al., 2017; Miller and Rogers, 2018; Jayasekera et al., 2020). No QPS about UE VCA has been created for people with UE amputations.

This study assessed the information needs of people with UE amputations and developed a UE VCA-specific QPS to foster patient-centered care. Our UE VCA-QPS was designed to help people with UE amputations attain greater information about UE VCA and establish realistic expectations through patient-provider communication to make informed decisions about UE VCA.

## Materials and methods

### Study design

In this cross-sectional study, we used a mixed-methods concurrent triangulation design (Greene et al., 1989; Creswell and Plano Clark, 2007), involving sequential in-depth and semi-structured interviews to develop and refine the UE VCA-QPS, as part of a larger study on decision making about UE VCA. Mixed-methods enabled elaboration and clarification of findings, increased validity of results, and informed subsequent data collection. We followed QPS development approaches used previously (Eggly et al., 2013; Ahmed et al., 2016; Lederer et al., 2016), and leveraged perspectives of patients and experts to ensure that the UE VCA-QPS is patient-centered.

The study was conducted at Northwestern University Feinberg School of Medicine (NU) in Chicago, IL, United States; Johns Hopkins University School of Medicine (JHU) in Baltimore, MD, United States; and Walter Reed National Military Medical Center (WRNMMC) in Bethesda, MD, United States from January 2020 through March 2022. Shirley Ryan AbilityLab in Chicago, IL, United States and David Rotter Prosthetics, LLC in Joliet, IL, United States supported recruitment for NU. The study was approved by the Institutional Review Boards at: NU (STU00209718), JHU (00225728), and WRNMMC (WRNMMC-EDO-2020-0432). NU served as the Institutional Review Board of record for WRNMMC. We used the Consolidated Criteria for Reporting Qualitative Research for quality reporting of qualitative studies (Tong et al., 2007).

### Participants and recruitment

Eligible participants were English-speaking adults age 18–65 years with acquired UE amputations who had not yet pursued UE VCA, UE VCA candidates (i.e., individuals who contacted a transplant center to express interest in pursuing UE VCA), UE VCA participants (i.e., individuals who began UE VCA evaluation), and UE VCA recipients. People who were cognitively impaired, and/or had congenital limb loss were excluded.

Multiple techniques were employed to increase sample size and ensure a representative sample (Patton, 2015). We recruited patients from study sites by mailing and/or emailing eligible individuals a letter describing the study, followed by a phone call a week later to screen for eligibility. Research team members did not have prior established relationships with study participants. We also recruited participants through support groups (*n* = 304) and social media websites (Facebook and Reddit) by emailing or posting study flyers online. Interested individuals contacting the team were screened by phone for eligibility. All participants provided verbal informed consent.

### Phase 1: Question prompt sheet item development

#### Data collection

In Phase 1, we conducted telephone in-depth interviews from July 2020–March 2022 to assess study participants’ information needs and questions about UE VCA. We drew upon five open-ended questions from the in-depth interview guide, which assessed: UE VCA information needs (“If you were thinking about getting an upper limb transplant, what would you want to know about it?” and “If you were thinking about getting an upper limb transplant, what information would you need?); UE VCA-related questions (“If you were thinking about getting an upper limb transplant, what questions would you have about it?”); and perceptions about a UE VCA-QPS (“What do you think about the QPS idea?” and “Would [the QPS] be worthwhile?”). The subset of candidates, participants, and recipients were also asked what people seeking UE VCA should be informed about (“What questions should people seeking an upper limb transplant ask about it to become well informed?” and “Could you suggest some things about upper limb transplantation that people with amputations should be informed about?”).

Interviews assessed study participants’ likelihood of using a UE VCA-QPS (on a 5-point Likert scale anchored by “Not At All,” “A Little,” “Somewhat,” “A Lot,” and “Completely”); demographics (e.g., age, gender); clinical background (e.g., date of amputation, amputation level and type); and health literacy (“How often do you need to have someone help you when you read instructions, pamphlets, or other written material from your doctor or pharmacy?” anchored by “Never,” “Rarely,” “Sometimes,” “Often,” and “Always”; “Never” and “Rarely” responses reflected adequate health literacy) (Morris et al., 2006).

Interviews were conducted by female and male research team members (BK, KV, MD, MN) trained by the Principal Investigator (EJG), a seasoned qualitative researcher, to ensure consistent and high quality data collection. Telephone cognitive interviews were conducted (by BK) with five participants prior to in-depth interviews (January–March 2020) using standard “think aloud” procedures to ensure interview guide questions were interpreted as intended and improve question wording (Singleton and Straits, 2017). Research team members took field notes during and after interviews. Interviews lasted, on average, 78 (range: 37–140) minutes and were audio-recorded and transcribed. Participants were compensated with a $35 gift card.

#### Content analysis

To identify potential QPS items, two members of the research team at each study site reviewed each transcription and compiled participants’ responses about UE VCA information needs and questions into a single document using content analysis (Bernard and Ryan, 1998). Responses were grouped into topics organized in terms of a patient’s progress from initiating evaluation to rehabilitation. All interview transcripts were then re-examined by research team members as individual files (within-case) and as a list of all participant responses to each open-ended question (across-cases) (Ayres et al., 2003). The Principal Investigator then reviewed responses under each topic to derive draft QPS items that synthesized the information needs and questions raised by all participant responses. Thereafter, the research staff reviewed the draft QPS items to: (a) confirm that they fully represented all participants’ responses, (b) add or delete repetitive or idiosyncratic items, and (c) revise item wording for clarity. This iterative process ensured comprehensiveness. All items were compiled into a first QPS draft comprising 130 items organized into 18 topics.

#### Thematic analysis

To identify UE VCA information needs, all transcripts were analyzed for themes emergent from the data using constant comparison, inductive, and deductive coding methods (Lincoln and Guba, 1985; Bradley et al., 2007). The research team established an initial codebook by developing deductive codes based on questions asked in the interview guide (e.g., Information Needs). The team then developed inductive codes based on themes emergent from the data during open coding of six transcripts until reaching thematic saturation (Miles and Huberman, 1994; Giacomini and Cook, 2000). Thereafter, two research team members at each study site (JG-S, BK, MD, KV, MN, ML) independently coded transcripts from their site using the finalized codebook in NVivo (Release 1.6.1, QSR International) until reaching inter-rater reliability (Cohen’s Kappa > 0.80) (Guest et al., 2011). Then, all transcripts were re-coded. During this process, the two research team members at each site resolved coding discrepancies through discussion. Finally, research team members reviewed all text segments coded as “Information Needs” to identify patterns and themes in study participants’ UE VCA information needs and developed code summaries (Keith et al., 2017).

### Phase 2: Question prompt sheet item refinement and reduction

#### Initial item refinement and reduction

Upper extremity VCA-QPS item reduction was performed by three research team members (EJG, BK, JG-S) by identifying redundancy and combining or removing redundant items to retain items that best conveyed the ideas. We assessed the readability of each item using the Flesch-Kincaid grade level formula (Stossel et al., 2012; Centers for Medicare & Medicaid Services [CMS], 2010), and simplified complex words and long sentences in items above a 6th grade reading level (Houts et al., 2006; Brega et al., 2015). Some items remained above a 6th grade reading level because they included terms that could not be restated in a simpler way or included three or more syllables (e.g., transplantation, anti-rejection, recipient). Additionally, we assessed the understandability and actionability of the UE VCA-QPS by applying the Patient Education Materials Assessment Tool (PEMAT) (Shoemaker et al., 2013). Following item refinement and reduction, the draft QPS included 77 items categorized into 16 topics.

#### Multidisciplinary review

**FIGURE 1:**
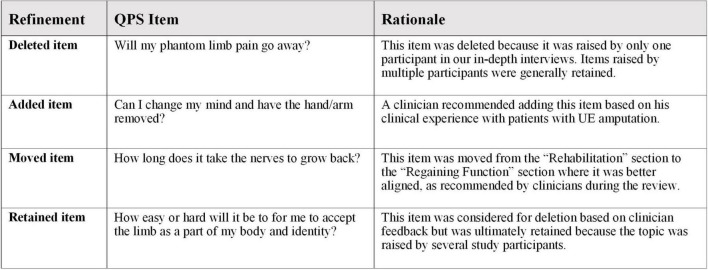
Examples of interim UE VCA-QPS refinement based on multidisciplinary feedback and research team review.

A 7-person multidisciplinary team of study collaborators comprised of researchers, UE VCA clinicians/surgeons, hand reconstructive surgeons, and occupational therapists reviewed the draft UE VCA-QPS and provided feedback on clinical accuracy, relevance to the UE VCA transplant evaluation process, clarity of question wording, and redundancy in question content. Based on the feedback, the research team revised item wording for clarity, moved items to different topic categories, combined items that addressed similar concepts, added items, and deleted items. Figure 1 includes examples of changes made to items and the rationale, based on multidisciplinary team feedback and research team review. Thereafter, the preliminary UE VCA-QPS included 52 items categorized into 12 topics.

#### Data collection

Semi-structured telephone interviews were conducted from September 2021– March 2022 by female and male research team members (BK, MD, WA, SF), trained by the Principal Investigator (EJG), to refine and reduce the 52-item preliminary UE VCA-QPS. Participants were asked to rate whether each question should be included in the UE VCA-QPS list on a 4-point Likert scale anchored by 4–“Definitely Keep,” 3–“Probably Keep,” 2–“Probably Cut,” and 1–“Definitely Cut.” Larger scores indicated a preference for item retention. When rating each question, participants were asked to consider how valuable each question and its answer would be to them if they were interested in the option of UE VCA. Closed-ended questions assessed participants’ likelihood of using the UE VCA-QPS if they were considering getting a UE VCA (on a 5-point Likert scale anchored by “Not At All,” “A Little,” “Somewhat,” “Very,” and “Completely”) and demographic and clinical characteristics (e.g., age, gender, amputation type and level, date of amputation). Open-ended questions assessed perceptions of the clarity of each item and suggestions for improving item wording, preferences between similar items, opinions about item order, and recommendations for adding or deleting questions. Interviews averaged 70 (range: 40–120) minutes and were audio-recorded, while research team members took field notes. Participants were compensated with a $35 gift card.

#### Mixed-methods analysis

Frequencies and means for each item’s Likert score were generated and reviewed. All items with a mean score of 3.00 or smaller were eliminated, resulting in 11 items cut from the list. Remaining items’ scores and qualitative responses were analyzed together. Participants’ qualitative responses were reviewed by the research team (EJG, JG-S, BK) to identify QPS item changes (e.g., rewording items, combining items, moving items). After refining QPS items, the draft QPS included 38 items categorized into 12 topics.

We then sought feedback on this UE VCA-QPS draft from the study’s Scientific Advisory Board (SAB), comprised of four bioethicists, and from the research team’s Co-Investigators, who included two UE VCA clinicians/surgeons and two hand surgeons, for further refinement. The SAB and Co-Investigators provided feedback on improving item wording and identifying items to combine, delete, or add. Further, the SAB and Co-Investigators were asked to ensure that items covered perspectives of people with UE amputations, the military, VCA ethics, UE VCA clinical care, hand surgery, rehabilitation, prosthetics, and disability rights. These steps aimed to ensure that the UE VCA-QPS would be relevant and meaningful for patient-provider discussions about UE VCA. Demographics and clinical characteristics of participants in the in-depth and semi-structured interviews were analyzed through descriptive statistics using IBM SPSS Statistics (Version 27).

## Results

### Demographics and clinical characteristics

Overall, 89 people with UE amputations participated (63.9% participation rate) in in-depth interviews (*n* = 50, 61.7% participation rate) and semi-structured interviews (*n* = 56, 65.9% participation rate) (Supplementary Figure 1). Seventeen individuals participated in both the in-depth interview and the semi-structured interview (NU: *n* = 4, JHU: *n* = 8, WRNMMC: *n* = 5). Sixty refused to participate before or after providing consent because they were not interested, too busy, compensation was too low, or they did not show up for their scheduled interview. Study participants included people with UE amputations who had not pursued UE VCA (85%), UE VCA candidates and participants (9%), and UE VCA recipients (6%). Most participants were male (73%), White (74%), on average 46 years of age, and had a unilateral amputation (84%) and had a below elbow amputation (56%) (Table 1). Participants were interviewed, on average, 10 years after their amputation. One-third (34%) did not use a prosthesis(es).

**TABLE 1:** Participants’ demographic and clinical characteristics.

Variable	Total (*n* = 89)	NU (*n* = 31)	JHU (*n* = 28)	WR (*n* = 30)
	*N* (%)	*n* (%)	*n* (%)	*n* (%)
**Age, years, mean [SD] (range)**	46.2 [10.9] (19–65)	50.0 [9.9] (25–65)	46.6 [10.0] (32–64)	41.9 [11.4] (19–65)
**Gender**				
Male	65 (73.0)	20 (64.5)	18 (64.3)	27 (90.0)
Female	24 (27.0)	11 (35.5)	10 (35.7)	3 (10.0)
**Ethnicity**				
Not Hispanic or Latino	80 (89.9)	29 (93.5)	27 (96.4)	24 (80.0)
Hispanic or Latino	9 (10.1)	2 (6.5)	1 (3.3)	6 (20.0)
**Race**				
White	66 (74.1)	23 (74.2)	23 (82.1)	20 (66.7)
Black or African American	16 (18.0)	5 (16.1)	5 (17.9)	6 (20.0)
Other*	7 (7.9)	3 (9.7)	0 (0.0)	4 (13.3)
**Marital Status**				
Married/Domestic Partner/Civil Union	57 (64.0)	19 (61.3)	16 (57.1)	22 (73.3)
Never Married/Single	17 (19.1)	5 (16.1)	8 (28.6)	4 (13.3)
Separated or Divorced	14 (15.7)	7 (22.6)	3 (10.7)	4 (13.3)
Widowed	1 (1.1)	0 (0.0)	1 (3.6)	0 (0.0)
**Education**				
Less than high school graduate	1 (1.1)	0 (0.0)	1 (3.6)	0 (0.0)
High school graduate	17 (19.1)	5 (16.1)	7 (25.0)	5 (16.7)
Some college	26 (29.2)	8 (25.8)	6 (21.4)	12 (40.0)
College graduate	27 (30.3)	12 (38.7)	6 (21.4)	9 (30.0)
Post graduate degree	18 (20.2)	6 (19.4)	8 (28.6)	4 (13.3)
**Employment Status** ^†^				
Employed Full time	37 (41.6)	11 (35.5)	11 (39.3)	15 (50.0)
Disabled	20 (22.5)	10 (32.3)	8 (28.6)	2 (6.7)
Retired	19 (21.3)	4 (12.9)	4 (14.3)	11 (36.7)
Employed Part time	4 (4.5)	2 (6.5)	2 (7.1)	0 (0.0)
Not Employed	4 (4.5)	3 (9.7)	1 (3.6)	0 (0.0)
Homemaker	3 (3.4)	1 (3.2)	1 (3.6)	1 (3.3)
Student	1 (1.1)	0 (0.0)	0 (0.0)	1 (3.3)
**Income**				
Less than $15,000	7 (7.9)	5 (16.1)	2 (7.1)	0 (0.0)
Between $15,000 and $34,999	7 (7.9)	1 (3.2)	5 (17.9)	1 (3.3)
Between $35,000 and $54,999	10 (11.2)	5 (16.1)	2 (7.1)	3 (10.0)
Between $55,000 and $74,999	13 (14.6)	5 (16.1)	4 (14.3)	4 (13.3)
Between $75,000 and $94,999	8 (9.0)	2 (6.5)	1 (3.6)	5 (16.7)
More than $95,000	36 (40.4)	12 (38.7)	12 (42.9)	12 (40.0)
Prefer not to answer	8 (9.0)	1 (3.2)	2 (7.1)	5 (16.7)
**Primary Health Insurance** ^‡^				
Medicaid or Medicare	41 (46.1)	16 (51.6)	14 (50.0)	11 (36.7)
Private	36 (40.4)	14 (45.2)	15 (53.6)	7 (23.3)
Uniformed Services (Tricare)	27 (30.3)	0 (0.0)	4 (14.3)	23 (76.7)
None	1 (1.1)	1 (3.2)	0 (0.0)	0 (0.0)
Other	2 (2.2)	1 (3.2)	1 (3.6)	0 (0.0)
**Health Literacy, Adequate**				
	80 (89.9)	30 (96.8)	24 (85.7)	26 (86.7)
	*N* (%)	*n* (%)	*n* (%)	*n* (%)
**Health Status** ^†^				
Excellent	18 (20.2)	6 (19.4)	6 (21.4)	6 (20.0)
Very good	36 (40.4)	11 (35.5)	14 (50.0)	11 (36.7)
Good	24 (27.0)	10 (32.3)	4 (14.3)	10 (33.3)
Fair	10 (11.2)	4 (12.9)	3 (10.7)	3 (10.0)
Poor	0 (0.0)	0 (0.0)	0 (0.0)	0 (0.0)
**Dominant Hand Before Amputation** ^†^				
Right	78 (87.6)	30 (96.8)	23 (82.1)	25 (83.3)
Left	8 (9.0)	1 (3.2)	3 (10.7)	4 (13.3)
**Upper Limb Amputated** ^†^				
Right	43 (48.3)	10 (32.3)	11 (39.3)	22 (73.3)
Left	31 (34.8)	13 (41.9)	12 (42.9)	6 (20.0)
Both	14 (15.7)	8 (25.8)	5 (17.9)	1 (3.3)
**Amputation Type**				
Unilateral	75 (84.3)	23 (74.2)	23 (82.1)	29 (96.7)
Bilateral	14 (15.7)	8 (25.8)	5 (17.9)	1 93.3)
**Amputation Level**				
Below elbow	50 (56.2)	19 (61.3)	12 (41.9)	19 (63.3)
Above elbow	37 (41.6)	11 (35.5)	15 (53.6)	11 (36.7)
Both below and above elbow	2 (2.2)	1 (3.2)	1 (3.6)	0 (0.0)
**Current Prosthesis Type** ^‡^				
Myoelectric	39 (43.8)	10 (32.2)	7 (25.0)	22 (73.3)
Mechanic	36 (40.4)	18 (58.0)	1 (3.6)	17 (56.7)
Cosmetic	4 (4.5)	1 (3.2)	1 (3.6)	2 (6.7)
Other	1 (1.1)	0 (0.0)	0 (0.0)	1 (3.3)
None	28 (31.5)	7 (22.6)	19 (67.9)	2 (6.7)
**Years Since First**^§^ **Amputation**				
<1 year	8 (9.0)	3 (9.7)	3 (10.7)	2 (6.7)
1–2 years	14 (15.7)	5 (16.1)	4 (14.3)	5 (16.7)
3–6 years	20 (22.5)	12 (38.7)	7 (25.0)	1 (3.3)
7–10 years	16 (18.0)	5 (16.1)	5 (17.9)	6 (20.0)
>10 years	31 (34.8)	6 (19.4)	9 (32.1)	16 (53.3)
**Data Collection Activity** ^**^				
In-Depth Interviews	50 (56.2)	16 (51.6)	17 (60.7)	17 (56.7)
Semi-Structured Interviews	56 (62.9)	19 (61.3)	19 (67.9)	18 (60.0)
**Type of Participant**				
Person with UE amputation	76 (85.4)	29 (93.5)	17 (60.7)	30 (100.0)
VCA candidate/participant	8 (9.0)	2 (6.5)	6 (21.4)	0 (0.0)
VCA recipient	5 (5.6)	0 (0.0)	5 (17.9)	0 (0.0)

SD, standard deviation; WR, Walter Reed National Military Medical Center.

*“Other” included people who identified as Hispanic or Mexican (n = 4), Asian (n = 1), Native Hawaiian or Other Pacific Islander (n = 1), or multi-racial (n = 1).

^†^Percentages do not add up to 100 because some participants did not respond.

^‡^Percentages add up to greater than 100 due to more than one response from some participants.

^§^Some participants had multiple surgeries for their amputation or multiple amputations.

**Some participants (n = 17) took part in both the in-depth interview and the semi-structured interview.

### Upper extremity vascularized composite allotransplantation information needs

When asked what information they would need if they were to pursue UE VCA, study participants reported different types of information that were organized into five major themes: broad and contextual information, information about the pre-UE VCA period, risks of UE VCA, the UE VCA procedure, and information about the post-UE VCA period. The major themes and subthemes are described below, with illustrative representative quotations presented in Table 2.

**TABLE 2:** Representative illustrative quotations about information needs by major theme and subtheme, with code frequency.

Themes/Subthemes	Code Freq. N	Quotations
**Broad and Contextual Information about UE VCA**		
Everything about it	8	“Well, I think I would need [to know] everything about it, like soup to nuts, like, oh, every aspect” [J008, 43-year-old female with bilateral below-elbow limb loss]
		
		“I hate to say this, but ‘everything’. [Laughs] That everything would include all of the risks, all of the benefits, and the projected recovery time as well as the actual success rates and actual recovery times of other patients.” [WR001, 52-year-old male with unilateral below-elbow limb loss]

UE VCA history and current state	13	“I’d like to know the kind of history of it, how did we get to the point where this is possible, how many transplants have been done.” [J012, 54-year-old female with unilateral below-elbow limb loss]
		
		“What I would want to know: the history, the research that was done…” [WR017, 57-year-old male with unilateral below-elbow limb loss]
		
		“The cost. If my insurance covered it. My out-of-pocket procedure costs.” [N017, 60-year-old female with unilateral above-elbow limb loss]

**Pre-UE VCA**		
Eligibility, waiting list, and evaluation process	13	“How one becomes a candidate first of all… how do you even get on their radar? And then how, what’s the process to find out if I’m a good candidate.” [N009, 53-year-old male with bilateral below-elbow limb loss]
		
		“How long does the process take as far as like, “OK, hey. We received a transplant. We need you here.” OK, so how would I get there?” [N015, 37-year-old female with unilateral below-elbow limb loss]
		
		“If I’m a good candidate, if my case is a good idea for the surgery?” [WR010, 35-year-old male with unilateral above-elbow limb loss]

Donor and matching process	14	“I would be curious about like where it is coming from, like this donor, and so I don’t know how much of that information they would share, but if I was getting somebody’s limb, I guess I probably would want to know who it was coming from.” [J014, 53-year-old female with unilateral above-elbow limb loss]
		
		“What’s the process, as far as choosing aesthetically where the arm comes from? How do they try to match up somebody, how do you get paired with somebody to actually have a transplant from their arm?” [WR008, 39-year-old male with unilateral above-elbow limb loss]

Transplant team and transplant clinic	9	“What is the background of the doctor? What are some of his cases, the number of surgeries, or the hospital staff that are involved and their experience with this? Ideally, I’d want to talk to a patient who’s had it done by this doctor. … what hospitals are doing it.” [N014, 58-year-old male with unilateral below-elbow limb loss]
		
		“How many has the doctor done? How many years [experience] do they have doing it? What experience do they have?” [WR011, 65-year-old male with unilateral above-elbow limb loss]

**Risks of UE VCA**		
Risks in general	10	“The risks that would be involved and if that would outweigh the, you know, if the reward would outweigh the risk.” [N011, 59-year-old male with unilateral below-elbow limb loss]
		
		“What are the risks to my health, what are the possibilities of what could go wrong or not happen for me or things like that.” [N022, 47-year-old female with unilateral below-elbow limb loss]

Effect on life and lifespan	10	“Would there be the possibility of me dying if I did this… if it rejects, what is the possibility of me dying from that…?” [J014, 53-year-old female with unilateral above-elbow limb loss]
		
		“And how does this change my long-term picture? Like, is this going to affect my lifespan? Is this going to shorten my lifespan at all?” [N019, 57-year-old male with unilateral below-elbow limb loss]

Infection and Rejection	7	“What happens if your body rejects the hand. You know, if you find out it wasn’t a match or something, do you have to take the hand off?” [WR017, 57-year-old male with unilateral below-elbow limb loss]

**UE VCA Procedure**		
Surgical procedure	15	“What will be done to my hand, or to my arm. Like, they’re going to. what they’re going to attach to what.” [N006, 47-year-old male with unilateral above-elbow limb loss]

		“I’d want to know all the ins and outs of the procedure itself, first, beforehand, like all the medical risks, the possible things that could happen.” [J014, 53-year-old female with unilateral above-elbow limb loss]

		“I would like to know how the surgery would go. How long would the surgery take? How do they attach it to the upper extremity?” [WR009, 62-year-old male with unilateral above-elbow limb loss]
Hospital stay and recovery period	17	“How long would I be incapacitated, like away from my home, away from my family.” [N017, 60-year-old female with unilateral above-elbow limb loss]
		
		“What the recovery process is, how long I would be spending in inpatient?” [WR003, 31-year-old male with unilateral above-elbow limb loss]

**Post-UE VCA**		
***Living with a UE VCA***		
Rehabilitation	26	“Where am I going to do my occupational therapy? Do I have to move from my home and live near the hospital because something might go bad… have to do that for 6 months?” [N019, 57-year-old male with unilateral below-elbow limb loss]
		
		“It’s like ‘How much time does that take out of your life? Would I have to go there and do all my rehab there? How does that work?.’ The amount of rehab, how many times a week, how long each time is, where the rehab is and about how long you’re doing rehab…” [J006, 64-year-old female with unilateral above-elbow limb loss]
		
		“I guess kind of the timeline as well and how much I would have to commit to physical therapy and occupational therapy. I guess more so just the timeline. Like when would you start seeing results, when should you be able to start moving like the elbow and those types of things.” [WR013, 24-year-old male with unilateral above-elbow limb loss]

Medication and medication side effects	17	“How many medications are there? Is this medication something that I have to take for the rest of my life? Is this once a week, twice a week, monthly, daily? …if I miss the time, will my body automatically reject the transplant?” [N015, 37-year-old female with unilateral below-elbow limb loss]
		
		“What does it mean to have some sort of medicine that’s in you that’s going to hurt you?” [J005, 40-year-old male with unilateral above-elbow limb loss]
		
		“The first and foremost thing I would want to know is how much medication I would have to take, and for how long.” [WR002, 39-year-old male with unilateral above-elbow limb loss]

Lifestyle changes	11	“What do I, do I have to readjust my diet from—way of eating, taking in certain foods? Do I, can I drink alcohol? Can I smoke tobacco?” [N007, 48-year-old male with bilateral, above- and below-elbow limb loss]
		
		“… what things might having a transplanted limb make you not able to do in terms of donating blood or in terms of just some ways that that might limit some of your choices going forward.” [J012, 54-year-old female with unilateral below-elbow limb loss]
		
		“I would need to know what … the environments that I may be precluded from taking part in, such as swimming, or working outside – how physically active can I be, and what environmental restrictions will that put upon me as to keeping me from doing things that I currently do.” [WR001, 52-year-old male with unilateral below-elbow limb loss]

***Outcomes of UE VCA***		
Functionality, sensation, and other outcomes	34	“You know, am I going to feel like a regular person again with two fully functioning arms? Or am I gonna have one good arm and a half dead arm. Where it’s still in the way and not very useful. I just went through all this work and all this procedure and all this surgery for something that I’m not even using still. Which is you know, kind of the problem with prosthetics.” [WR003, 31-year-old male with unilateral above-elbow limb loss]
		
		“I would like to know if, if I will be able to use my hand like my hand was. Will I be able to, as a female, paint the fingers…the fingertips? How will I be able to use it functionally, like being able to use a keyboard going back to work? And for me, the biggest thing is, can I use it to do hair?” [WR005, 56-year-old female with unilateral below-elbow limb loss]
		
		“Is this hand transplant, is it going to replace what I lost? And if not, what percentage will I reacquire, you know? … What is my percentage of recovery? That’s what I would want to know.” [N021, 56-year-old male with bilateral below-elbow limb loss]
		
		“How is the functionality of an arm transplant? If you have an arm transplant, does it end up being just as functional as your own arm?” [J006, 64-year-old female with unilateral above-elbow limb loss]

Success rate	12	“The surgeries that have been done, … how many have turned out wonderful, how many are OK, and how many are not OK.” [N018, 63-year-old male with unilateral above-elbow limb loss]
		
		“I guess the percentages of successful transplant and the non-successful transplants, and the rate of rejection.” [WR017, 57-year-old male with unilateral below-elbow limb loss]

Experiences of UE VCA recipients	13	“I’d like to hear what experience people have with it from medical professionals, but also, hopefully or possibly by people who have had transplant of an upper extremity who can at least talk about their experience.” [J012, 54-year-old female with unilateral below-elbow limb loss]
		
		“I think I would want to know the experience of other individuals… What is it they weren’t able to do with a prosthetic, let’s say, but now they can do, or things that were maybe a little bit more difficult with a prosthetic but now they can do it with ease.” [N022, 47-year-old female with unilateral below-elbow limb loss]
		
		“And [I’d] probably like to talk to people [the doctors] have had—you know they have worked on… helped with. Because it is a lot easier for the doctors or for people to say well it’s supposed to work like that, but the guy that’s actually feeling it, the guy that actually has it, he can kind of tell you the real deal, you know.” [WR011, 65-year-old male with unilateral above-elbow limb loss]
		
		“The information I would need is, I’d want to know that someone else had had the procedure and had had success with it. I’d want to talk to that person, hear from that person.” [WR007, 41-year-old male with unilateral below-elbow limb loss]

#### Broad and contextual information

Participants desired broad and contextual information about UE VCA that included knowing “everything” about it as well as the historical context and present status of UE VCA. Knowing “everything” about UE VCA would enable participants to gain “a more in-depth understanding.” Participants desired historical information including “the research that was done,” and how the transplant field got “to the point where this is possible.” The current status of the UE VCA field pertained to “how many people have had the procedure done” and “the current state of technology.” Additionally, only participants at NU wanted to know about the “upfront and lifetime costs” of UE VCA and the insurance coverage and out-of-pocket expenses.

#### Pre-upper extremity vascularized composite allotransplantation

Several participants wanted information about the processes occurring prior to the UE VCA procedure. This information encompassed the eligibility criteria for UE VCA (e.g., “what would make a good qualified patient…”), the waiting list (e.g., “how long of a wait [would there be] on the waiting list for a set of arms to become available”), and the evaluation process (e.g., what does “the psychological review process… entail”). Participants also desired information about the donor process, such as “how long do you have to typically wait for a donor” and how well can they “match an arm to my body to make it look more realistic for myself.” Participants desired learning about the transplant team and clinic, such as the “doctors and clinicians [who have] done it and have had success,” “how many procedures they have done,” and “where it would be taking place.”

#### Risks of upper extremity vascularized composite allotransplantation

Participants wanted information about the risks of UE VCA, including general risks and the potential affect UE VCA could have on one’s life. General UE VCA risks included “the possibilities of what could go wrong” from receiving a UE VCA. Participants desired information on how UE VCA could potentially harm a person’s life and lifespan, including the “rate of life-threatening risks,” the risk of dying, infection, rejection, and whether recipients would “be more susceptible to cancers or other things like COVID.”

#### Upper extremity vascularized composite allotransplantation procedure

Participants desired information about the UE VCA surgical procedure, including the hospital stay and recovery period. Desired procedure details included “how they attach the bone,” the required number of surgeries, and the “length of time the surgery usually takes.” Others wanted information about the length of hospital stay post-transplant, and the recovery process: “how long [the UE VCA] is going to take to heal.”

#### Post-upper extremity vascularized composite allotransplantation

Participants expressed interest in learning how UE VCA impacts a recipient’s life as it relates to medication and medication side effects, rehabilitation, function, success rate, lifestyle changes, and experiences of UE VCA recipients. Regarding medication and medication side effects, participants desired information on “how many drugs you have to be on for the rest of your life” and the “risk levels of life-time, consistent use of drugs and side effects of the drugs.” Study participants also desired information on what “the rehab process [would] be like,” how long they would do hand therapy, and “how much time [it would] take out of [their] life.”

Participants were interested in learning about the type and extent of functionality a UE VCA would provide, whether recipients would “experience the sensation of touch again,” and how long it would take “to get it working.” Some referred to their previous “natural hand” and wanted to know if a UE VCA would “work like my old arm worked.” Participants framed their interest in learning about the success rate in terms of “how many [UE VCAs] failed versus how many succeeded.” Others wanted to learn about required lifestyle changes, such as how long recipients might be “out of work” and any restrictions on diet, drinking alcohol, and smoking. Several participants reported interest in learning directly from UE VCA recipients about their experiences: “what kind of things can, and can’t they do.”

### The final question prompt sheet

The final UE VCA-QPS has 35 items, organized into 9 main topics, and fits onto one double-sided page of paper. At the end of the list, patients can note additional questions. The UE VCA-QPS is available upon request.

Mean ratings of QPS items in the semi-structured interviews ranged from 2.70 to 3.93. Items ranked largest, reflecting preference for retention, were primarily about UE VCA risks. Items ranked moderately high were about UE VCA functional outcomes.

### Likelihood of using the question prompt sheet

Among in-depth interview participants, most who were asked (*n* = 36/45, 80%) reported being “Completely” or “A Lot” likely to use a QPS (Table 3). Among all semi-structured interview participants, most (86%) reported being “Completely” or “Very” likely to use a QPS.

**TABLE 3:** Likelihood of using a UE VCA-QPS.

Question*	Not at all likely *n* (%)	A little likely *n* (%)	Somewhat likely *n* (%)	A lot/very likely^†^ *n* (%)	Completely likely *n* (%)
**In-Depth Interviews** If you were considering getting an upper limb transplant, how likely would you be to use a question prompt sheet? *n* = 45^‡^	1 (2.2)	4 (8.9)	4 (8.9)	6 (13.3)	30 (66.7)
		
**Semi-Structured Interviews** If you were considering getting an upper limb transplant, how likely would you be to use a question list about hand or upper limb transplantation in your doctor visit? *N* = 56	4 (7.1)	0 (0.0)	4 (7.1)	18 (32.1)	30 (53.6)

*Some participants (n = 17) took part in both the in-depth interview and the semi-structured interview.

^†^One anchor in the rating scale differed between the two types of interviews: “A Lot Likely” was used in the in-depth interview and “Very Likely” was used in the semi-structured interview.

^‡^n = 5 participants were not asked the question.

## Discussion

Through mixed-methods research, we developed a 35-item QPS specific to UE VCA to address the information needs of people with UE amputations and facilitate patient-centered care. Our study participants had extensive information needs, focusing on risks, the rehabilitation process, and expectations for functional and other outcomes of UE VCA. The UE VCA-QPS is intended for use in the UE VCA clinical context amongst candidates and participants undergoing UE VCA evaluation. Study participants reported a high likelihood of using the UE VCA-QPS in a clinic visit if they were to pursue UE VCA. The UE VCA-QPS supports patient-centered care by promoting patient-provider communication that addresses patients’ unique information needs and fosters information sharing so that patients can make informed treatment decisions.

People with UE amputations are typically healthy people, who generally have little need to know about transplantation. Our study participants had considerable information needs suggesting that they had limited knowledge of transplantation, rejection, and anti-rejection medications, which underscores the need to help people with UE amputations learn about the UE VCA option and establish realistic expectations so that they can make informed treatment decisions. The higher priority placed on risks and functional outcomes by participants in the semi-structured interviews indicates the relative importance that providers should emphasize in their discussions about UE VCA.

Our finding that only NU participants desired information about the costs of UE VCA makes sense considering that: (a) JHU participants included more UE VCA candidates, participants, and recipients and were thus more familiar with the insurance and out-of-pocket costs associated with UE VCA; and (b) all WRNMMC participants were military health system beneficiaries whose healthcare costs are covered by the federal government.

The UE VCA-QPS can be provided to patients in advance of their first visit to the VCA clinic, or it can be provided to them for review while waiting in the clinic to see their provider so that they can become more empowered to communicate with providers. Evidence shows a QPS may be more effective at increasing patient question-asking and provider information-giving when the QPS is provided to patients shortly before they meet with their provider (Sansoni et al., 2015). By reviewing the UE VCA-QPS prior to seeing the provider, the patient can identify questions they find important and become more engaged during their visit. Providers should ask patients for their QPS question list at the beginning of their visit given that other research has shown this provider practice of “endorsement” is effective at increasing the number of questions asked by patients and the amount of information provided by doctors during consultations (Sansoni et al., 2015).

In general, QPSs can vary in format and length. The number of items in other QPSs range from 3 to 169 items, with a mean of 33 items (Kinnersley et al., 2007; Brandes et al., 2015; Sansoni et al., 2015). Our 35-item UE VCA-QPS is comprehensive while also convenient in fitting onto a two-sided single page of paper for easy distribution, or may be viewed as an electronic document on a mobile phone, tablet, or computer.

Future research should assess the effectiveness of the UE VCA-QPS in facilitating communication between patients and providers in the UE VCA clinical context, as well as patients’ informed decision-making about UE VCA. Implementation science research should assess the most acceptable, appropriate, and feasible way of delivering and evaluating the UE VCA-QPS.

Strengths of our study include a multi-site study design conducted in geographically diverse locations in the US, and included individuals throughout the US. Our sample included civilian and military participants with unilateral and bilateral amputations that were above and/or below the elbow, which supports the transferability of findings, despite being a challenging population to recruit. Additionally, our sample is representative of the broader population of people with UE amputations in terms of gender, race, and age (Inkellis et al., 2018). Our mixed-methods design facilitated a patient-centered approach to QPS development by involving people with UE amputations in multiple phases of data collection, review, and feedback, and prioritizing their perspectives over other stakeholders’ feedback. Further, our multidisciplinary team of study collaborators included UE VCA clinicians/surgeons, hand reconstructive surgeons, and occupational therapists who helped to ensure that the UE VCA-QPS was clinically relevant for the target population. Designing the UE VCA-QPS at a low reading grade level and use of the PEMAT will foster a greater comprehensibility (Shoemaker et al., 2013).

Our study has limitations. Some (19%) participants completed both the in-depth and semi-structured interviews, which may reduce the transferability of study results. Although individuals motivated by the prospect of pursuing UE VCA may have been more inclined to participate in interviews, suggesting a selection bias, study participants’ views ranged broadly in their level of interest in pursuing UE VCA. As our study sample included disproportionately fewer Hispanic or Latino individuals compared to the U.S. population of people with UE amputations (10% versus 15%), future research should examine UE VCA information needs among more ethnically diverse participants. We produced the UE VCA-QPS in English; future research should prepare the UE VCA-QPS in other languages.

## Conclusion

People with UE amputations desired extensive information about UE VCA, primarily on risks, the rehabilitation process, and functional outcomes. To empower people with UE amputations and foster patient-provider communication about UE VCA, we developed a 35-item UE VCA-QPS. Use of the UE VCA-QPS is designed to address information needs, facilitate patient-centered care, and enhance informed decision making among people with UE amputations undergoing evaluation for UE VCA.

## Data availability statement

The original contributions presented in this study are included in the article/Supplementary material, further inquiries can be directed to the corresponding author.

## Ethics statement

The studies involving human participants were reviewed and approved by the Institutional Review Boards at: Northwestern University (STU00209718), Johns Hopkins University (00225728), and Walter Reed National Military Medical Center (WRNMMC-EDO-2020-0432). Northwestern University served as the Institutional Review Board of record for Walter Reed National Military Medical Center. Written informed consent for participation was not required for this study in accordance with the national legislation and the institutional requirements. All participants provided verbal informed consent.

## Author contributions

EJG conceived and designed the research study and participated in performing the research, analyzing and interpreting the data, and writing the manuscript. JG-S and BK participated in performing the research, analyzing and interpreting the data, and writing the manuscript. MD, KV, MN, and ML participated in data collection and analyzing and interpreting the data. TR, WA, and SF participated in data collection. All authors reviewed and approved the final manuscript.

## Supplementary material

Supplementary Figure 1 Consort diagrams for in-depth interviews and semi-structured interviews.

## Abbreviations

JHU: Johns Hopkins University
NU: Northwestern University
QPS: question prompt sheet
UE: upper extremity
VCA: vascularized composite allotransplantation
WRNMMC: Walter Reed National Military Medical Center.
