# Supplementary figures and images for: Information needs and development of a question prompt sheet for upper extremity vascularized composite allotransplantation: A mixed methods study
Source: Front Psychol. 2022 Sep 5;13:960373. doi: 10.3389/fpsyg.2022.960373 (PMC9484522; doi:10.3389/fpsyg.2022.960373)

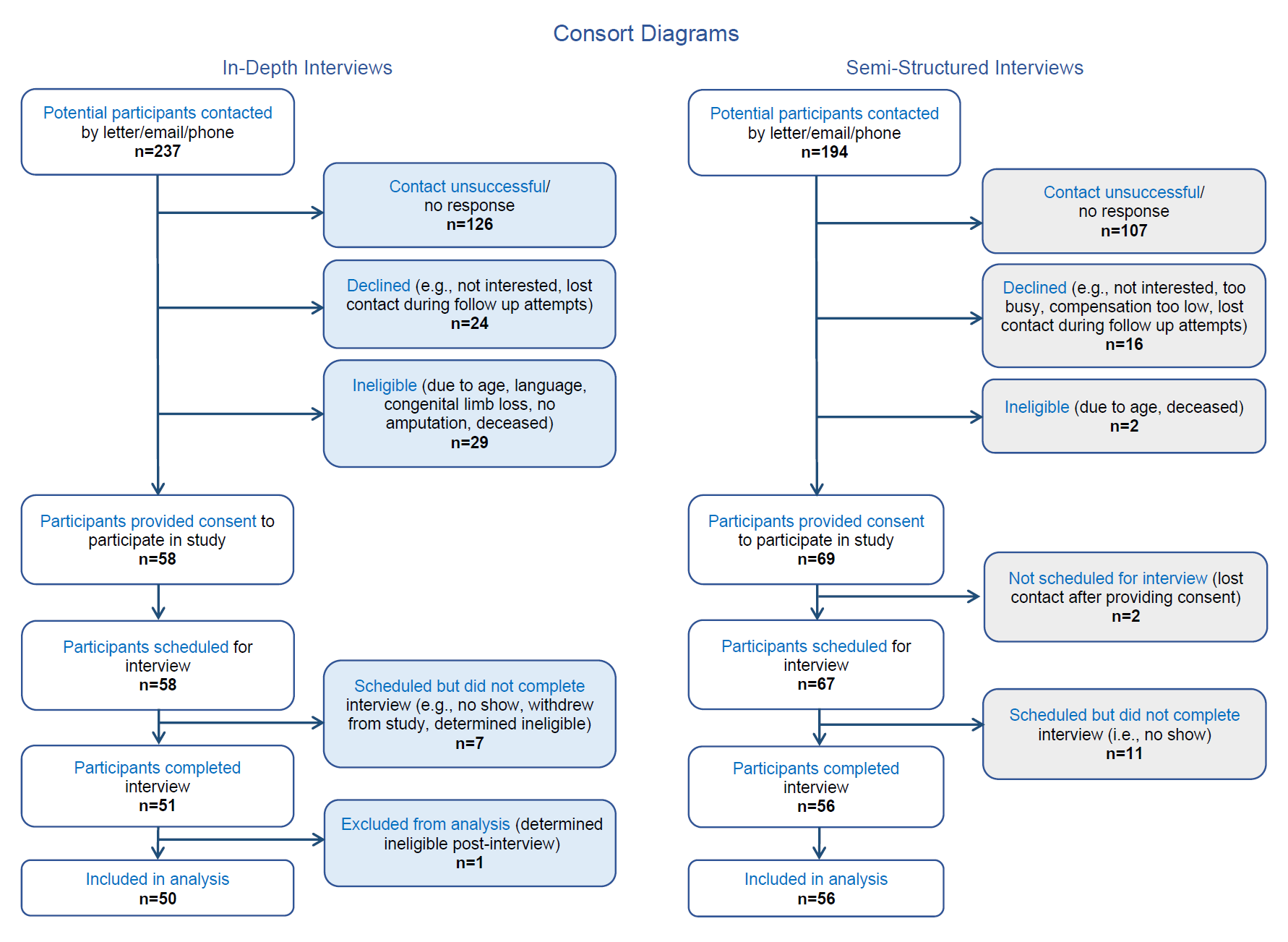

Supplement: Supplementary Figure 1 — Consort diagrams for in-depth interviews and semi-structured interviews. [file Image_1.png]
